# Intervening to reduce sedentary behavior in older adults – pilot results

**DOI:** 10.15171/hpp.2019.09

**Published:** 2019-01-23

**Authors:** Kelli F. Koltyn, Kevin M. Crombie, Angelique G. Brellenthin, Brianna Leitzelar, Laura D. Ellingson, Jill Renken, Jane E. Mahoney

**Affiliations:** ^1^Department of Kinesiology, University of Wisconsin-Madison, Madison, Wisconsin, USA; ^2^Department of Kinesiology, Iowa State University, Ames, Iowa, USA; ^3^Wisconsin Institute for Healthy Aging, Madison, Wisconsin, USA; ^4^Department of Medicine, University of Wisconsin School of Medicine and Public Health, Madison, Wisconsin, USA

**Keywords:** Sitting, Older adults, Sedentary intervention, Behavior change, Self-regulation, Physical function

## Abstract

**Background: ** Older adults spend most of their day in sedentary behavior (SB) (i.e., prolonged sitting), increasing risk for negative health outcomes, functional loss, and diminished ability for activities of daily living. The purpose of this study was to develop and pilot test an intervention designed to reduce SB in older adults that could be translated to communities.

** Methods: ** Two pilot studies implementing a 4-week SB intervention were conducted. SB,physical function, and health-related quality of life were measured via self-report and objective measures. Participants (N=21) completed assessments pre- and post-intervention (studies 1 and 2) and at follow-up (4-weeks post-intervention; study 2). Due to the pilot nature of this research, data were analyzed with Cohen’s d effect sizes to examine the magnitude of change in outcomes following the intervention.

**Results:** Results for study 1 indicated moderate (d=0.53) decreases in accelerometry-obtained total SB and increases (d=0.52) in light intensity physical activity post-intervention. In study 2,there was a moderate decrease (d=0.57) in SB evident at follow-up. On average SB decreased by approximately 60 min/d in both studies. Also, there were moderate-to-large improvements in vitality (d=0.74; study 1) and gait speed (d=1.15; study 2) following the intervention. Further,the intervention was found to be feasible for staff to implement in the community.

**Conclusion: ** These pilot results informed the design of an ongoing federally funded randomized controlled trial with a larger sample of older adults from underserved communities. Effective,feasible, and readily-accessible interventions have potential to improve the health and function of older adults.

## Introduction

It is well known that insufficient physical activity is a risk factor for numerous chronic diseases and premature mortality.^1,2^ More recently, sedentary behavior (SB) has been identified as a health risk that is additional to, and distinct from, too little exercise. SB defined as sitting or reclining during waking hours with a low energy expenditure^3^ has emerged as a new focus for intervention research versus the more traditional approach of increasing moderate-to-vigorous intensity physical activity. Accumulating evidence indicates a link between SB and adverse health outcomes, independent of physical activity.^4^ In other words, even if an individual exercises every day, the amount of time they sit affects future health.

Older adults represent one of the fastest growing segments of the population and spend 60%-70% of their waking hours in sedentary activities, increasing their risk for negative health outcomes.^5^ Specifically, greater sedentary time is associated with an increased risk of functional decline, chronic disease, and premature mortality. Emerging research indicates that breaks in sedentary time (i.e., standing up) are associated with better health and function in older adults.^6^ Thus, interventions that shift the focus from increasing exercise to breaking up extended sitting time by standing up and moving more throughout the day may improve the health of older adults, but there is limited research examining such interventions. Several intervention studies (primarily single-group pre-post design) with older adults have shown decreases in SB ranging from 9 to 132 minutes, with larger reductions for self-reported SB as opposed to objectively measured SB.^7-12^ These studies provide preliminary support that SB can be reduced immediately following an intervention in older adults but additional research is needed, specifically to assess whether reductions in SB are sustained after an intervention ends. Additionally, it is important to understand the impact of SB interventions on specific health domains (e.g., physical function, physical activity levels, and quality of life) in addition to SB outcomes.

Therefore, with funding from the Greater Wisconsin Agency on Aging Resources, we conducted preliminary research to develop a translational community-based intervention to reduce SB in older adults. In collaboration with the Rock County Council on Aging, two pilot studies (study 1 and study 2) were conducted in Rock County, WI which is made up of small urban and rural communities. The purpose of this research was to examine the effectiveness of the intervention to reduce SB, as well as examine the feasibility of delivering the intervention in a community setting, with a specific focus on acceptability, implementation, practicality, and integration into the community.^13^

## Materials and Methods

This work represents the initial steps in the development and testing of an intervention intended to be implemented and sustained by State Aging Units in community settings (Note: Aging Units offer evidence-based health promotion programs to older adults in their counties through funding from the Older Americans Act [OAA]). When a new health promotion program is shown to be evidence-based (i.e., effective, translatable), Aging Units can use OAA funding to support and sustain implementation of the program). Translational evidence-based programs have the potential to result in sustained programming to improve the health and function of older adults in the community setting.

The design of the two studies was a pre-post design. Study 1 involved developing the intervention and testing it with a small sample of older adults in a community setting. Our community partners, the Greater Wisconsin Agency on Aging Resources (GWAAR) and the Rock County Council on Aging, were instrumental in the design and testing of the intervention. GWAAR provides community-based aging services to 70 counties and 11 tribes in Wisconsin and considers interventions to increase physical activity a high priority area. For this reason, GWAAR funded our preliminary research, recruited the Rock County Council on Aging to work with us, and was a key resource in the development of the intervention curriculum. The intervention, based on self-regulation theory, was delivered as a workshop (i.e., sit less) and focused on eliciting ideas from older adults regarding how they could reduce their sitting time, helped them set practical goals and develop action plans to reach them, and refined their plans across sessions to promote sustainable behavior change. The intervention consisted of four weekly 1.5 hour sessions and was delivered by the Director of the Rock County Council on Aging.

### Study 1 methods

Older adults ≥ 65 years were recruited by the Director to participate in a 4-week behavior change intervention designed to break up SB by standing up multiple times throughout the day. An introductory session was held in which participants signed an informed consent document and then completed a Demographic and Health History questionnaire and the Short Form–36 (SF-36) questionnaire, a widely used and validated measure of health-related quality of life, that consists of eight subscales (e.g., vitality, physical functioning, general health, bodily pain, physical role functioning, emotional role functioning, social functioning, and mental health).^14^ Participants were interviewed regarding their SB over the past week using a validated questionnaire.^7^ The questionnaire asked about SBs during the past week including time spent watching television/movies, computer use, reading, socializing, transportation, hobbies, working (for pay or volunteer), and any other sedentary activities not included in the preceding categories. Next, participants were issued two monitors, the Actigraph^™^ GT3X+ accelerometer (Actigraph, LLC, Fort Walton Beach, FL) and the activPAL^™^(PAL Technologies Ltd, Glasgow, UK) to objectively measure physical activity and SBs. The Actigraph (worn on the hip) was used to classify physical activity behaviors into different intensities (e.g., light, moderate, vigorous). The activPAL3 (worn on the thigh) was used to assess body positions (e.g., standing vs sitting/lying down). Participants were directed to wear both monitors during waking hours, unless bathing or swimming, for a 7-day period. The monitors were returned one week later.

The intervention was delivered via a small group workshop format. Research suggests the ideal range to facilitate active group participation is 10-15 participants^15^ to create an environment with enough people to have diversity in sharing yet few enough to ensure that all participants can be involved. Strategies incorporated into the workshop sessions included information dissemination, individual goal setting, development of action plans, self-monitoring, group discussions, and various problem solving activities. Through structured activities, the trained facilitator elicited participants’ reasons for prolonged sitting and motives for sitting less (e.g., improved functioning, i.e., ability to get up out of a chair), supported participants’ choices (autonomy) of short-term goals, and engaged them in making weekly action plans to sit less. Participants were shown how to appropriately set and adjust goals and to self-monitor their behavior by completing brief daily logs at the end of each day. Older adults were asked to break up prolonged sitting (one hour or more) by standing up an extra 3–5 times/day progressing up to 10–12 times/day by the fourth week. At the end of session 4, participants again completed the SF-36 and were interviewed about their SB over the past week using the same questionnaire that was used previously. Participants were then issued the Actigraph and activPAL monitors to wear for another 7-days, and monitors were returned one week later. Participants also completed a brief questionnaire asking them about their satisfaction/perception of the intervention workshop and were given a $25 gift card for participating in this study.

### Study 2 methods

Study 2 was a follow-up to study 1 and included an additional assessment at 8 weeks to examine whether changes in SB persisted after the intervention ended. The same 4-week workshop was delivered by the Director of the Rock County Council on Aging; however, to facilitate ongoing behavior change (i.e., reductions in SB after the workshop ended), a refresher session was held at 6 weeks with the follow-up assessment at 8 weeks. In addition, an objective measure of physical function was included in study 2. The Short Physical Performance Battery (SPPB)^16^ consists of a balance test, a 4-meter walk for usual gait speed, and a timed measure of chair stands. Adults ­>65 years of age were recruited to participate in study 2. Participants completed the same questionnaires as in study 1 (i.e., Demographic & Health History and the SF-36), the SPPB, were interviewed about their time spent in sedentary activities over the past week, and wore the Actigraph and activPAL monitors for one week before and after (i.e., 4 & 8 weeks) the intervention workshop. Participants received a $25 gift card for their participation in study 2. In addition, the Director of the Rock County Commission on Aging was interviewed to gain insight into the feasibility of delivering the intervention in a community setting.

### Data processing

Data from the Actigraph and activPAL monitors were processed together using the validated Sojourns Including Posture method, which combines acceleration and postural data from the two monitors, and subsequently uses an artificial neural network to identify bouts of activity based on rapid acceleration/deceleration/postural changes, and assigns MET values to each bout.^17^ See Ellingson et al^17^for additional information regarding activity monitoring processing using the Sojourns Including Posture method. In healthy adults, 3-4 days of monitor wear has been shown to capture about 80% of the inter-individual differences in activity levels.^18,19^ Thus, inclusion criteria for both the Actigraph and activPAL were a minimum of 10 hours per day wear time, on at least 4 days. Sixty or more minutes of no movement (zero acceleration in all 3axes) was considered non-wear time and excluded from further processing.

### Data analyses

Due to the preliminary nature of this pilot study and small group format, Cohen’s *d* effect size calculations (defined as the difference between means, M1–M2, divided by the pooled standard deviation) were used to characterize the magnitude of change in SB, health-related quality of life, and physical function after participation in the intervention.^20^ Interpretation of Cohen’s *d* effect sizes allows for the magnitude of the treatment effect to be classified as small (*d* = 0.20 to 0.49), moderate (*d* = 0.50 to 0.79), or large (*d* = 0.80 and above). {Note: results reported below are for variables with moderate to large effect size changes. Additional outcomes collected but not reported in this short communication primarily yielded small effect size changes}.

## Results

### Study 1 results

Twelve older adults (10 women) with a mean age of 69 years (SD = 4.5) participated in this study. The demographic and health information of these participants are summarized in Table 1.

### Sedentary Behavior & Physical Activity

**Figure 1:**
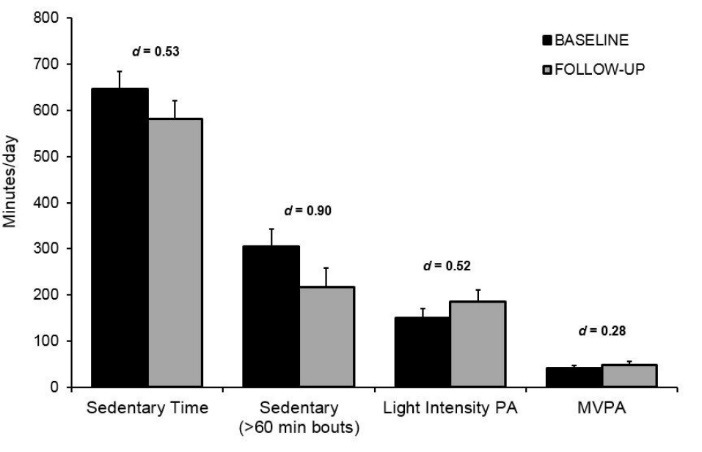
Objectively measured sedentary time, light-intensity, and moderate-to-vigorous physical activity (MVPA) at baseline and post intervention for study 1. Error bars depict standard error.

*Objective measures (monitors):* The participants in this study had high levels of SB at the outset averaging almost 11 h/d (mean = 647 min/d; SD = 124). Following the 4-week intervention workshop, there was a moderate decrease (*d* = 0.53) in total sedentary time (mean = 582 min/d; SD = 118) with the time spent sitting/reclining decreasing by over one hour/day (i.e., 65 min/d). In addition, there were large reductions (*d *= 0.90) in prolonged bouts of SB (i.e., sitting ≥ 60 minutes at a time) which are thought to be the most detrimental to health.^21^ Moreover, there were moderate increases (*d *= 0.52) in light intensity physical activity and small increases (*d *= 0.28) in moderate-vigorous physical activity (MVPA) after the 4-week intervention workshop. On average, participants increased their physical activity by 41 min/d (light intensity = 35 min/d and MVPA = 6 min/d). These data are illustrated in Figure 1.

*Self-Reported Measure of Sedentary Behavior:* Participants self-reported spending approximately 11 h/d in SB (mean = 678 min/d; SD = 314) before the workshop began with a large self-reported decrease (*d *= 0.95) after the 4-week workshop (mean = 446 min/d; SD = 174). The three sedentary activities reported being engaged in most often were watching TV/videos; reading; and socializing with moderate self-reported reductions in these activities following the workshop (*d *= 0.50; 0.56; 0.43 respectively). These data are summarized in Table 2.

*Other Outcomes:* Results indicated there were moderately large improvements in vitality (*d *= 0.74) and self-reported general health (*d *= 0.72) with a small-to-moderate improvement in physical function (*d *= 0.47) as measured by the SF-36. Strategies used most often by participants to reduce SB included standing up during TV commercials, standing up while reading, and spreading household chores out across the day. Participants reported that breaking up sitting time was more appealing to them than increasing exercise.

### Study 2 results

**Figure 2:**
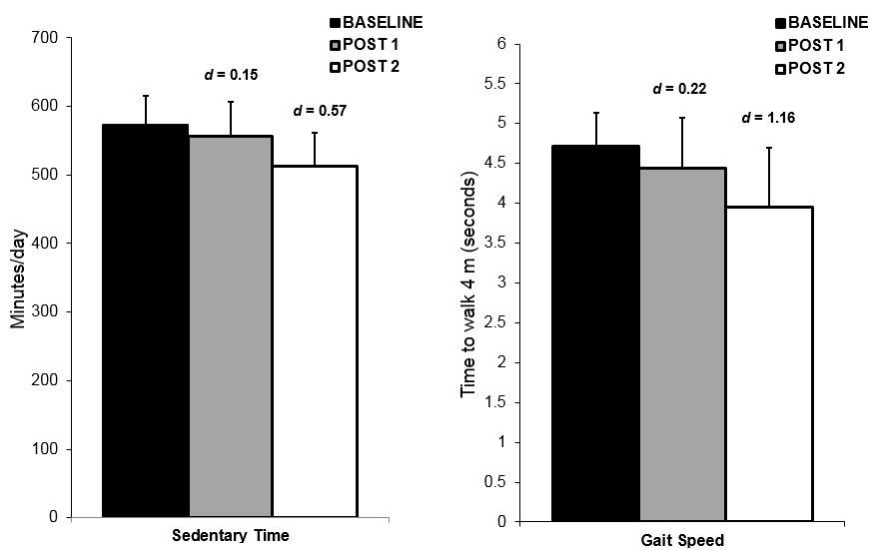
Objectively measured sedentary time and gait speed at baseline, post intervention (post 1) and follow-up (post 2) for Study 2. Error bars depict standard error.

Nine older adults (7 women) with a mean age of 68 years (SD**= 8) participated in study 2. The demographic and health information of these participants are summarized in Table 1. The aim of this follow-up study was to examine whether changes in SB would be evident/sustained after the intervention workshop ended (i.e., at the 8-week follow-up). Prior to the workshop, participants spent approximately 9.5 h/d (mean = 574 min/d; SD = 102) engaged in SB. Immediately following the workshop, there was a small decrease in (*d *= 0.15) in accelerometry-obtained total sedentary time (mean = 557 min/d; SD**= 122) with a moderate reduction (*d *= 0.57) in total sedentary time at follow-up (mean = 513 min/d; SD = 107; see Figure 2). On average, total sedentary time decreased by approximately 60 min/d which was evident 4-weeks after the workshop ended (i.e., at the 8-week follow-up) indicating that changes in SB occurred post-intervention. For self-reported SB, there were moderate-to-large decreases in total sedentary time at 4-weeks (*d *= 0.49) and follow-up (*d *= 0.77). The sedentary activities with the largest decreases at follow-up included sitting at the computer (*d *= 0.78), while socializing (*d *= 0.74), and doing hobbies (*d *= 0.74). Further, there was a large effect size improvement (*d *= 1.15) in gait speed at the follow-up assessment (see Figure 2). Strategies used most often to reduce SB included standing up during TV commercials, standing up while reading or talking on the phone, spreading household chores out across the day, and putting the TV remote farther away.

In addition, the Director of the Rock County Commission on Aging indicated delivering the intervention in a community setting was feasible (e.g., “The intervention worked because it was simple. It is not complex and that is the beauty of it”). The Director as well as the participants in the studies indicated satisfaction with the intervention (i.e., acceptability), and the intervention was described as practical (i.e., easy for the Director to deliver and for the participants to complete). Also, the Director indicated the intervention would be a good fit to include in health programming for older adults in her county (i.e., integration).

## Discussion

Although SB interventions have been found to be feasible to implement in other populations (e.g., younger adults),^22^ only recently have investigations begun to examine the feasibility and efficacy of administering interventions in older adults, especially to those who might experience greater barriers accessing the intervention (e.g., rural communities). In summary, there is a recognized need for feasible, effective interventions that can be disseminated into real-world community health programs for older adults. The intervention to reduce SB examined in this research was developed in collaboration with community partners, and was specifically designed for older adults to make small incremental changes to their daily routines. The intervention showed promise of being successful (both effective and feasible). The results indicated the intervention reduced SB by approximately 60 min/d and was associated with moderate increases in light intensity physical activity. These results could have important public health implications since Buman et al^23^ reported that replacing 30 min/d of SB with equal amounts of light intensity physical activity was associated with better health in older adults. Also, the large effect size improvements in mobility (i.e., gait speed) and vitality suggest that decreasing SB could be an innovative strategy to improve physical function and well-being in older adults. Importantly, reductions in SB were evident 4 weeks following delivery of the intervention. Furthermore, the intervention was found to be feasible for staff to implement (i.e., acceptable, easy to deliver, practical, and could be integrated into community health programming). Partnering with community aging organizations is advantageous because it capitalizes on social networks in the community as well as existing resources providing an opportunity to offer the intervention on a continuing basis.

As is often the case with pilot research, there are several limitations that need to be addressed with future trials. For instance, sample size was limited in both studies, and the vast majority of the sample consisted of women. Moreover, neither study 1 nor study 2 implemented a control or comparison group. Despite the aforementioned limitations, these promising results provided the groundwork for a randomized controlled trial with a larger sample of older adults. Funding has been provided by the National Institutes of Health to examine translating a “Stand Up and Move More” intervention by State Aging Units to older adults in underserved communities. This ongoing statewide study is a collaboration with community partners in four counties (i.e., rural and African American communities) to determine the feasibility and effectiveness of this intervention to reduce SB and improve physical function in older adults in underserved communities. In conclusion, sitting less seems simple, but intervention is necessary to get older adults to do it. If breaking up sitting time by standing up and moving more throughout the day proves to be effective, then we will have identified an innovative and practical intervention with the potential to improve the health and function of older adults that could be translated to communities across the country.

## Ethical approval

All procedures performed in studies 1 and 2 involving human participants were in accordance with the ethical standards of the institutional and/or national research committee and with the 1964 Helsinki Declaration and its later amendments or comparable ethical standards. All procedures were approved by the Social and Behavioral Sciences Institutional Review Board at the University of Wisconsin—Madison. Informed consent was obtained from all individual participants included in the study.

## Competing interests

The authors declare that they have no competing interests.

## Funding

This study was funded by the Greater Wisconsin Agency on Aging Resources and the University of Wisconsin Virginia Horne Henry Fund. In addition, the project was facilitated by the University of Wisconsin Community-Academic Aging Research Network (CAARN), through funding from the UW School of Medicine & Public Health, and from the Clinical & Translational Science Award (CTSA) program, through the NIH National Center for Advancing Translational Sciences (NCATS), grant UL1TR000427. The content is solely the responsibility of the authors and does not necessarily represent the official views of the NIH. The funding bodies had no role in the design of the study, and in the collection, analysis, interpretation of data, and in writing the manuscript.

## Authors’ contributions

KFK, JR, and JEM were involved in the conception and designing the study. KFK, KMC, AGB, LDE were responsible for data acquisition. KFK, KMC, AGB, BL, LDE were involved in data and statistical analyses. KFK, KMC, BL were involved in the initial manuscript preparation, and AGB, LDE, JR, and JEM helped to evaluate and edit the manuscript.

## Acknowledgments

We would like to thank Joyce Lubben for conducting the workshops, and Keith Gennuso for assisting with data collection.

**Table 1:** Demographic and health history information

	**Study 1**	**Study 2**
Age (y)	68.86 (4.53)	67.83 (7.73)
Height (cm)	162.10 (9.55)	160.23 (8.65)
Weight (kg)	88.05 (16.6)	86.18 (27.25)
BMI	33.92 (8.11)	33.18 (7.72)
Marital status (%)		
Single	11%	0%
Married	44%	0%
Divorced	11%	83%
Widowed	33%	17%
Educational attainment (% with college degree)	44%	50%
Racial/Ethnic background (% white)	100%	100%
Health History (% reporting yes)		
High blood pressure	88%	83%
Heart problems	33%	17%
Arthritis	78%	50%
Diabetes	33%	50%
Stroke	11%	0%
Cancer	11%	0%

Note. Values are mean (SD) unless otherwise noted.

**Table 2:** Self-reported sedentary behaviors from study 1

**Self-report measure (min/d)**	**Pre**	**Post**	**Effect Size (** ***d*** **)**
Watching TV/videos/DVDs	260.24 (259.97)	165.71 (121.40)	0.50
Computer/internet use	53.57 (84.85)	29.05 (67.07)	0.32
Reading	106.67 (114.59)	53.81 (73.22)	0.56
Socializing	99.52 (82.59)	71.43 (47.29)	0.43
Driving/riding in a vehicle	48.25 (41.32)	42.46 (36.37)	0.15
Hobbies	30.54 (37.04)	24.76 (26.66)	0.18
Work (paid or volunteer)	18.09 (32.19)	9.52 (28.57)	0.28
Other	70.65 (73.82)	49.68 (39.93)	0.37
Total	678.19 (314.15)	446.43 (174.03)	0.95

Note. Values are mean (SD) unless otherwise noted.
